# Being accurate about accuracy in verbal deception detection

**DOI:** 10.1371/journal.pone.0220228

**Published:** 2019-08-08

**Authors:** Bennett Kleinberg, Arnoud Arntz, Bruno Verschuere

**Affiliations:** 1 Department of Security and Crime Science, University College London, London, United Kingdom; 2 Department of Psychology, University of Amsterdam, Amsterdam, The Netherlands; Middlesex University, UNITED KINGDOM

## Abstract

**Purpose:**

Verbal credibility assessments examine language differences to tell truthful from deceptive statements (e.g., of allegations of child sexual abuse). The dominant approach in psycholegal deception research to date (used in 81% of recent studies that report on accuracy) to estimate the accuracy of a method is to find the optimal statistical separation between lies and truths in a single dataset. However, this method lacks safeguards against accuracy overestimation.

**Method & Results:**

A simulation study and empirical data show that this procedure produces overoptimistic accuracy rates that, especially for small sample size studies typical of this field, yield misleading conclusions up to the point that a non-diagnostic tool can be shown to be a valid one. Cross-validation is an easy remedy to this problem.

**Conclusions:**

We caution psycholegal researchers to be more accurate about accuracy and propose guidelines for calculating and reporting accuracy rates.

## Introduction

Verbal credibility assessment, or verbal deception detection, plays an important role in legal proceedings when physical evidence is absent or inconclusive. Courts often have to rely heavily on statement credibility analysis to ascertain whether a suspect’s testimony is to be believed, whether witness statements are credible, or whether children’s accounts of alleged sexual abuse are truthful or fabricated [1]. The recent case of allegations of sexual misconduct against US supreme court nominee Brett Kavanaugh illustrates the challenge. Kavanaugh was accused of a sexual assault more than 30 years before his nomination [2,3]. A Judiciary Committee hearing revolved around statements made by Kavanaugh and his accuser, requiring the committee to make an assessment largely on the basis of the statements made.

Several US federal agencies including the FBI, Army Military Intelligence, and the US Immigration and Naturalization Service have been trained in Scientific Content Analysis (SCAN, see http://www.lsiscan.com/id29.htm). Forensic psychologists and expert witnesses in legal proceedings in Germany, the UK and the Netherlands use Criteria-based Content Analysis (CBCA) as a means to determine the veracity of allegations of child sexual abuse [4][5]. A promising new tool for airport security screening is predominantly based on verbal indicators of deception [6] and there is ample evidence that verbal deception detection methods such as CBCA help distinguish between truthful and deceptive accounts better than chance [7,8]. While different in the exact scoring methods applied, all these statement credibility assessment methods share a standard procedure: suspects or witnesses are asked to provide a statement about an event, which is then transcribed and analysed by experts on a range of dimensions such as the level of detail of the statement. But how accurate are such verbal credibility assessment tools?

The typical way in which psycholegal deception researchers answer that question is by building a dataset consisting of truthful and deceptive statements coded on a set of verbal indicators such as the richness in detail and logical consistency. While there are diverse methods to assess the ability of the verbal criteria to differentiate truth-tellers from liars, researchers then tend to rely on linear discriminant analysis–a statistical technique that will provide the optimal function of verbal indicators to discern lie from truth [9]. Importantly, the predominant approach in verbal deception research is to build a discriminant function that separates truthful from deceptive statements on the same dataset that it is tested on. That procedure was used in 77% of studies (S1 Appendix) where researchers reported an accuracy of their approach (i.e. the percentage of truthful and deceptive statements identified as such). This figure has not changed: papers published between 2010–2017 show that 81% make use of that statistical procedure that we call the “training set optimisation technique”. By capitalising on idiosyncrasies of the dataset, this procedure diminishes its vital goal: assessing the ‘true’ accuracy of the classification if it were to make a credibility prediction for a novel, out-of-sample set of statements (see also [10]). The current paper examines that problem and discusses how methods that are standard practice in machine learning research can function as a safeguard against inflated accuracy rates, namely cross-validation and independent sample validation.

## Method and results

The code for the simulation studies and the resulting reproducible data are available at https://osf.io/2dcs5/files/.

### The current practice

Simulation procedure: We simulated data for a sample of *n* = 1,000 with 8 (as with a popular verbal approach used in research, Reality Monitoring, [8]), 12 (as for a popular verbal approach used in practice, SCAN, [11]), or 19 (as with CBCA, [8]) predictors. The correlation between the predictors and the binary outcome was held constant at either *r* = 0.0 (no relationship between predictors and binary outcome) or *r* = 0.1 (weak relationship between predictors and binary outcome). The latter was chosen to reflect the small effect sizes that are common in verbal deception research [12–14].

We iteratively simulated data for sample sizes of *n* = 40 up until *n* = 1,000 in steps of 10. For each step, we ran 100 simulations and averaged the classification accuracies. For each sample size, we calculated the accuracy obtained with the training set optimization method. The class membership of the binary outcome (i.e., proxies for truthful vs deceptive) was 50/50, and the priors in the latent discriminant analysis were set accordingly. The simulations were conducted using the caret [15] and MASS [16] packages in R [17]. The plots were created using ggplot2 [18].

### Findings

**Fig 1:**
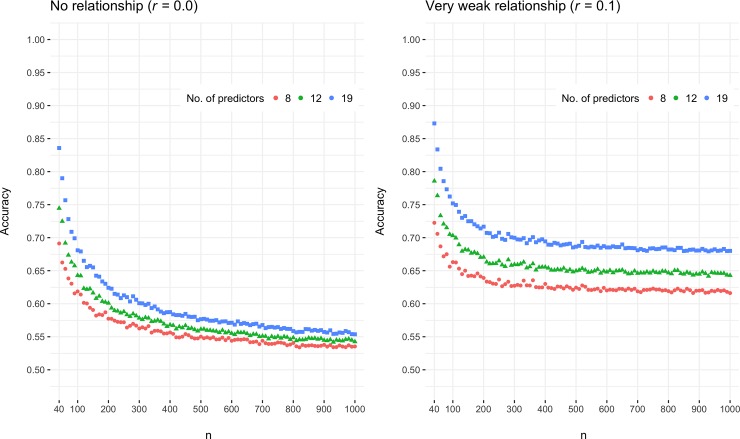
Accuracies for latent discriminant analysis without cross-validation on simulated data for 8, 12 and 19 predictors with increasing *n*. The averaged accuracy is displayed on the y-axis and the increased sample size for the simulation on the x-axis.

Fig 1 shows the volatility in the accuracy as estimated by latent discriminant analysis when using 8, 12, or 19 predictors. For the sample sizes typical for the field, *M*_*n*_ *= * 61, range_*n*_ = 10 to 240 in the most recent meta-analyses [4,7], the accuracy estimates are unreliable and display a highly volatile pattern. The weaker the diagnostic value of the verbal indicators and the more indicators used, the more problematic the erroneous accuracy estimates. In a sample of 40 participants, a completely undiagnostic verbal tool (*r* = 0.0) with 19 indicators can be estimated to have up to 84% accuracy. In sum, the dominant practice of using linear discriminant analysis on a single dataset provides highly inaccurate and overly optimistic accuracy estimates.

### Fixing the problem

A primer on cross-validation: The desirable evaluation of the predictive accuracy of a classification algorithm occurs when the algorithm is built on one dataset and tested on an entirely new dataset. The purpose of any classification is to make an inference about how accurately future cases could be assigned their correct outcome class (e.g. truthful or deceptive). However, is it always necessary to test a classifier on completely new, independent data and collect new data? Especially when high-quality data are hard to obtain (e.g., allegations of child sexual abuse—the primary usage of the CBCA) or labor-intensive (e.g., several trained human coders rate a large number of statements on a number of verbal criteria) it would be desirable to have a precise accuracy estimate derived from a single dataset. A compromise solution, then, is to treat the individual dataset as if it were multiple datasets [19].

Consider a study where the researchers collected 50 truthful and 50 deceptive statements. If the aim is to assess the performance of a deception detection method, the soundest manner would be to build a classification algorithm (e.g. a linear discriminant analysis, a logistic regression model, a support vector machine classifier) on these 100 statements and then collect new data to assess how well that classification algorithm performs on *unseen* data. This would mirror the actual potential application, namely assessing the veracity of a statements that were not used to inform the original classifier. When independent sample validation is not feasible, the researchers may want to resort to cross-validation.

In cross-validation, the data are split and recycled. For the purpose of this paper, we briefly introduce two kinds of cross-validation. In *k*-fold cross-validation, the data (here: 100 statements) are split into *k* folds, of which *k*-1 folds are used as training set and 1 fold is used as test set. For example, a 10-fold cross-validation would split the data into stratified folds of 10 statements (5 deceptive, 5 truthful). Then 9 folds (= 90 statements) are used as the training set to build a classification algorithm, and 1 fold (= 10 statements) is used to assess the accuracy of that classifier. This procedure is repeated until each fold has been used as test set at least once. Typically, the performance metrics (e.g. accuracy) are then averaged across the ten test set iterations [20].

Another kind of cross-validation is the leave-one-out method. Here, rather than splitting the data into folds, the classification algorithm is built on *n*– 1 data points (here: 99 statements) and is tested on the remaining, left-out data point. That procedure is repeated until each data point has been left-out once. In doing so the leave-one-out procedure recycles data even more than the *k*-fold procedure but is computationally costlier for vast sample sizes and models with large numbers of predictors. Since the current sample sizes in deception research are relatively small, we focus on leave-one-out cross-validation for the remainder of this paper.

In sum, cross-validation handles a part of the dataset as the model building dataset (or *training set*), and another part to assess its performance (*test set;* [20]). Analogous to the training-vs-test set terminology, the training set optimisation technique reports accuracies of the training set only, thereby lacking the critical evaluation of the predictions.

### Procedure

To examine how the current practice–the training set optimization technique–compares to cross-validation and independent sample validation, we simulated data for 19 predictors (as in CBCA) with a weak individual predictor-outcome relationship (*r* = 0.124, converted from Cohen’s *d* = 0.27, the average effect size for CBCA criteria in a recent meta-analysis [12]). CBCA was chosen because it is the tool most frequently used in forensic practice [1,21]. We calculated the prediction accuracies–for increasing sample sizes–obtained from linear discriminant analysis using both the training set optimisation technique and leave-one-out cross-validation. Both procedures resulted in a predictive model (i.e. empirically determined linear combinations of the predictors that separate the data into two classes–deceptive and truthful) that was then additionally validated on a novel, also simulated, test set of the same size (i.e., of the training set was *n* = 40, the test set also was *n* = 40).

### Findings

**Fig 2:**
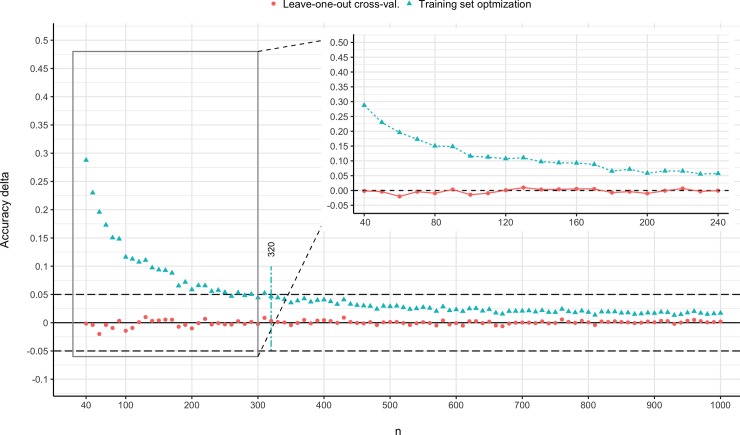
Accuracy differences (y-axis) between traditional training set optimisation and leave-one-out cross-validation compared to independent test set validation for increasing sample sizes (x-axis). The dashed horizontal grey lines indicate the upper and lower boundary of the [-0.05; +0.05] stability corridor. The vertical coloured line indicates the sample size points-of-stability for the training set optimisation technique. Inset plot: accuracy difference scores zoomed in for sample size between 40 and 240.

Fig 2 shows the differences between the accuracies yielded on the training set with the current practice of training set optimisation and leave-one-out cross-validation, and the independent test set. Values larger than zero indicate that the accuracy estimation method (here: either leave-one-out cross-validation or the training set optimization) produces values that are overestimations of the accuracy that you would obtain with a new, independent sample drawn from the same distribution. We adopted the points-of-stability procedure [22] to evaluate at which sample size, the fluctuation is deemed practically irrelevant. We defined a stability corridor of an accuracy difference of [+0.05; -0.05], that allowed us to find the point-of-stability in sample size *n* after which the accuracy difference compared to the test set evaluation does not leave the exceed the upper or lower boundary of the above-mentioned stability corridor. Fig 2 allows for the following conclusions:

- When relying on the dominant training set optimisation, the sample size needs to be substantially larger (*n* > 320) than is typical in the psycholegal literature (40 < = *n* < = 240) to eliminate accuracy overestimations.
- The overestimation of the accuracy achieved with the training set optimisation technique is substantial. This is especially the case for sample sizes commonly used in verbal credibility assessment research (40 < = *n* < = 240). For these sample sizes, the overestimation is on average 12 percentage points in accuracy (range: 6 to 29). For published papers which lack cross-validation, this graph can be used as an approximate estimate of the magnitude of inflation in the reported accuracy. For example, for a study with 80 participants without cross-validation, the accuracy might need to be corrected downwards with at least 12 percentage points (e.g., reported accuracy = 75%; corrected estimate = 63%). Note that such accuracy corrections imply that one simplifies the verbal deception detection tool by aggregating the effect sizes of multiple indicators and by not considering potential moderators. Our findings should be interpreted as the needed correction *on average* rather than as a generalization to individual studies. Nevertheless, the point remains that a lack of any kind of validation results in overoptimistic accuracies unrepresentative of the *true* underlying accuracy of the tool in question.
- Leave-one-out cross-validation safeguards against accuracy overestimation. Cross-validated predictive models fare consistently well when validated on a test set; the accuracy difference never exceeded 5% even for small sample sizes.

### The importance of cross-validation: Illustration with real verbal credibility assessment data

To illustrate the importance of validation outside of the simulation scenario, we obtained the raw data from recently published verbal deception detection studies. The two datasets were about truthful and deceptive statements of someone’s recent negative autobiographical event, manually annotated with SCAN (original data published in [22,23]). In the original studies, the authors compared SCAN, a tool popular with practitioners yet in the academic community heavily criticized for having low reliability and validity [21,24,25], with other verbal credibility tools and concluded that it is inferior to other tools and lacks validity. For illustrative purposes, we re-analysed the data provided for SCAN with and without cross-validation. We chose SCAN as a re-analysis example to illustrate the problem of the current procedure that may incorrectly yield evidence in support of a widely debunked method [21,24,25]. Table 1 shows that linear discriminant analysis on the original dataset without cross-validation (= current practice) would misleadingly suggest SCAN to be able to discern a lie from the truth. As shown above, cross-validation can protect from accuracy inflation. With leave-one-out cross-validation, it becomes clear that SCAN did not perform better than chance. It is troubling that the dominant practice would present the heavily criticised and non-substantiated tool SCAN [21,24,25] as a valid method (i.e. better than chance accuracy) to classify lies and truths.

**Table 1:** Illustration how the dominant practice (linear discriminant analysis with training set optimisation) can lead to an erroneous conclusion.

	Training set optimisation (current practice)	Leave-one-out cross-validation (recommended practice)
Accuracy estimate	61.54% [54.98–67.80]	51.28% [44.68–57.85]
Conclusion	Significantly better than chance classification.	No better than chance classification.

## Discussion

Reviewing the literature on verbal credibility assessment, we note that the vast majority of studies that report accuracy rates try to find the *optimal* statistical separation between deceptive and truthful statements through verbal criteria within a single dataset. Through simulations, we show that this technique leads to imprecise accuracy estimates for the sample sizes that are typical for the verbal deception detection field. Accuracy estimates are systematically overestimated for *n* < 320. Our simulations suggest that the accuracies reported in the majority of the published verbal deception detection research may need to be corrected downwards between 6 and 29 percentage points (average: 12 percentage points), depending on sample size, due to a suboptimal statistical classification procedure. Such a correction is substantial considering that the average accuracy rates of verbal deception research as a whole only exceed the random guessing baseline by about 20 percentage points [7,8].

### Suggestions for remedy

Our proposed solution is three-fold (Table 2). First, at the minimum, we recommend using cross-validation for all prediction algorithms. Cross-validation comes at practically no cost, is provided by all statistical software packages (e.g., SPSS, R, JASP), and ensures a leap in accuracy precision. Note that cross-validation is standard practice in computational disciplines using machine learning. Therefore, we are not introducing cross-validation as a new concept, nor are we the first to advocate the use of cross-validation as a safeguard against inflated accuracy estimates in deception research (see e.g., [26]) but it remains infrequently used as of to date. However, given the far-reaching implications of verbal deception research in practice and the lack of any cross-validation in the majority of studies, we argue that the field as a whole would benefit from a gentle reminder to use better methods to estimate the accuracy of the methods used. It is worthwhile pointing out that cross-validation comes in many fashions and thereby leaves the exact choices to the researcher (e.g. one could use leave-one-out, split-half, holdout, or re-usable holdout cross-validation, to name but a few [20,27,28]). To avoid that cross-validation is tried repeatedly until favourable results are obtained—analogous to *p*-hacking in behavioural research [29]–one may opt to pre-register the cross-validation procedures; that is determining the procedure beforehand, making a public pre-registration of that procedure, and reporting the findings of that procedure [30].

**Table 2:** Suggestions for the improvement of the accuracy estimation in the predictive analysis in verbal credibility assessment research.

Remedy	Key Advantage	Key challenge	Safeguard
Validation on an independent sample	- Allows for robust claims regarding the generalizability of findings	Resource intensive (new data collection)	- Pre-registration of the classification algorithm - Sharing classification algorithm as a data file
Cross-validation	- Easy to implement (no new data collection needed, often default setting in statistical software)	- Might still capitalise on idiosyncrasies of the sample	- Pre-registration of cross-validation procedure
Larger sample sizes	- Solidifies conclusions on statistical inferences and prediction metrics	- Resource intensive	- Preregistration of sample size justification - Open sharing of data

Second, even preferable to cross-validation is testing the prediction algorithm on a new sample. Since cross-validation still relies on just one sample and because the essential test of a method is how well it performs on *unseen* data, researchers would want to assess a classifier algorithm derived from one data collection moment (e.g. one experiment) on freshly collected data (e.g. a second, identical experiment). Early findings on such independent sample validation in verbal deception research suggest that classification algorithms are less robust against sample variations than expected [31]. This independent sample validation would at the same time provide the much needed direct replications of verbal deception studies [32](for an exception see [33]). Independent sample validation ideally implies that the classifier used is pre-registered. However, because it is not always feasible to re-run an experiment, a fast and easy alternative is to share the classifier algorithm derived from a study with the community. Each classifier can be stored in a sharable data file using statistical software. By making the classifier available, other researchers can test how well it performs on their dataset.

Third, increasing sample sizes. Such a call for larger sample sizes has repeatedly been made [19,34] and we think that our current demonstration may help convince verbal deception researchers of its importance. The sample sizes of verbal deception research are small, and the public availability of research data is still scant. This leaves a void of large datasets that are needed to assess the methods of the field reliably. We strongly encourage the research community to conduct studies with larger sample sizes. More specifically, we call for the verbal deception detection community to embrace practices that directly address the logistical impediments of collecting data from large samples, such as Study Swap (https://osf.io/view/studyswap/), the Many Labs initiative (e.g., [35]) and Registered Reports [36], as well as the Psychological Science Accelerator [37]. With a move towards better research practices, in particular in psychological research, we agree with Yarkoni and Westfall that “[in] many cases there is a serious debate to be had about whether it is scientifically useful to conduct small-sample research at all” [19].

## Conclusion

Verbal credibility assessment and its scholarly backcloth of verbal deception research remain of vital importance in legal settings, and it is not foreseeable that this will change in the nearby future. Therefore, it should be in the interest of the general public, the scholarly community and practitioners that the reporting of the diagnostic ability of verbal deception detection methods meets the highest standards. The psycholegal deception research community has just made the first steps towards critical self-reflection and more research transparency [38,39]. We hope that this paper adds to the debate and encourages verbal credibility research to become more accurate about its accuracy.

## Supporting information

S1 Appendix (DOCX)
