# Supplementary material for: Being accurate about accuracy in verbal deception detection
Source: PLoS One. 2019 Aug 8;14(8):e0220228. doi: 10.1371/journal.pone.0220228 (PMC6687387; doi:10.1371/journal.pone.0220228)
Supplement: S1 Appendix — (DOCX) [file pone.0220228.s001.docx]

### Supporting information 1

### Appendix

**Literature review**

To provide a contemporary overview of the state of classification in human coded verbal deception detection, we reviewed all published papers used in the most recent meta-analyses in the field (1–4) that reported accuracy rates, used verbal indicators to discriminate between truthful and deceptive statements, and relied on experimental data collection. Of these 169 papers, 56 reported classification analyses - 41 of which (73%) did not use some (cross-)validation.

The meta-analyses do not include the most recent work on the topic. To explore whether research practices have changed more recently, we also inspected all recent (last five years; 2012-2017), English, peer-reviewed papers on human coded verbal credibility assessment. We searched the *Web of Science* database on the search terms (1) “Criteria-based content analysis”, “CBCA”, “reality monitoring”, “RM”, “scientific content analysis”, ”SCAN”, “statement validity assessment”, “SVA”, “validity checklist” (connected by OR) combined with “psychology of evidence”, “statement analysis”, “credibility”, “credibility judgment”, “deception” (connected by OR). After excluding overlapping papers already contained in the three meta-analyses, this resulted in an additional 20 papers of which 9 reported classification analyses - 8 of which (89%) did not use (cross-) validation. The detailed list of all included papers and their annotation is available at <https://osf.io/9psek/>.

References to the appendix:

1. Hauch V, Sporer SL, Masip J, Blandón-Gitlin I. Can credibility criteria be assessed reliably? A meta-analysis of criteria-based content analysis. Psychol Assess. 2017;29(6):819–34.

2. Hauch V, Blandón-Gitlin I, Masip J, Sporer SL. Are computers effective lie detectors? A meta-analysis of linguistic cues to deception. Personal Soc Psychol Rev [Internet]. 2015 [cited 2017 Jul 6];19(4):307–342. Available from: http://journals.sagepub.com/doi/abs/10.1177/1088868314556539

3. Oberlader VA, Naefgen C, Koppehele-Goseel J, Quinten L, Banse R, Schmidt AF. Validity of Content-Based Techniques to Distinguish True and Fabricated Statements: A Meta-Analysis. Law Hum Behav. 2016;40(4):440–457.

4. Vrij A, Fisher RP, Blank H. A cognitive approach to lie detection: A meta-analysis. Leg Criminol Psychol. 2017 Feb;22(1):1–21.
